# Mitochondrial Translation Inhibition Triggers an Rst2-Controlled Transcriptional Reprogramming of Carbon Metabolism in Stationary-Phase Cells of Fission Yeast

**DOI:** 10.3390/biom15101354

**Published:** 2025-09-24

**Authors:** Ying Luo, Shaimaa Hassan, Saniya Raut, Jürg Bähler

**Affiliations:** 1 Institute of Healthy Ageing, Department of Genetics, Evolution & Environment, University College London, London WC1E 6BT, UK; ying.luo@ucl.ac.uk (Y.L.); shaimaa.hassan.18@ucl.ac.uk (S.H.); saniya.raut.23@ucl.ac.uk (S.R.); 2 Jiangsu Key Laboratory for Microbes and Genomics, School of Life Sciences, Nanjing Normal University, Nanjing 210023, China

**Keywords:** *S. pombe*, retrograde response, carbon catabolite repression, Scr1, transcription factor, transcriptome, mitochondrial metabolism, stress response, RNA-seq, genetic interactions

## Abstract

Mitochondria possess their own genome, which encodes subunits of the electron transport chain, rendering mitochondrial protein translation essential for cellular energy metabolism. Mitochondrial dysfunction affects nuclear transcription through the retrograde response. We applied RNA-seq to investigate whether and how the inhibition of mitochondrial translation by chloramphenicol (CAP) affects transcriptome regulation in proliferating or stationary-phase cells of *Schizosaccharomyces pombe* growing in fermentative or respiratory media. Stationary-phase cells in glucose medium exhibited the strongest transcriptome response to CAP, characterized by expression signatures similar to those observed under other stresses, including the retrograde response. The induced genes were also significantly enriched in cytoplasmic carbon metabolism pathways, reflecting a transcriptional reprogramming from respiration to fermentation. The transcription factors Scr1 and Rst2, regulators of carbon catabolite repression (CCR), controlled a common set of carbon metabolism genes in CAP-treated stationary-phase cells, and they showed opposing effects on the lifespan of these cells. Rst2 was required for the induction of carbon metabolism genes and maintained nuclear localization in CAP-treated stationary-phase cells. A systematic genetic interaction screen revealed functional relationships of Rst2 with processes related to stress and starvation responses. These findings uncover a complex transcriptional program in stationary-phase cells that adapt to inhibited mitochondrial translation, including stress- and retrograde-like responses, contributions of the CCR factors Scr1 and Rst2, and adjustment of carbon metabolism to deal with mitochondrial dysfunction.

## 1. Introduction

Mitochondria are double-membrane-bound organelles in most eukaryotic cells that are primarily responsible for energy production through the oxidative phosphorylation (OXPHOS) system. In addition to their role in cellular bioenergetics, mitochondria are involved in a wide range of important biological processes, including the regulation of intermediary metabolism, calcium homeostasis, ferroptosis, apoptosis, and aging [1,2,3]. Mitochondria possess their own genome (mtDNA), which encodes several essential proteins required for the assembly and function of the electron transport chain (ETC). In the fission yeast *Schizosaccharomyces pombe* (*S. pombe*), the mtDNA is ~19 kb in size and encodes seven key subunits of the ETC (*cytb* of complex III; *cox1, cox2,* and *cox3* of complex IV; *atp6*, *atp8*, and *atp9* of ATP synthase), two rRNAs (*rns* and *rnl*), one small subunit of ribosomal protein (*rps3*), and a full set of tRNAs [4].

The expression of mtDNA-encoded proteins relies on a dedicated mitochondrial translation machinery, which is more closely related to the bacterial counterpart than to the cytosolic one, reflecting the endosymbiotic origin of mitochondria [5,6]. Unlike bacterial translation initiation, which requires three essential initiation factors (IF1, IF2, and IF3), mitochondrial translation initiation typically involves only two initiation factors, mtIF2 and mtIF3 [7,8]. Mammalian mtIF2 proteins contain a unique 37-amino-acid insertion domain that exhibits functional similarities to bacterial IF1 and is required for efficient mitochondrial translation in vitro [9]. *S. pombe* contains two mitochondrial translation initiation factors, Mti2 (mtIF2) and Mti3 (mtIF3), with Mti2 playing a more critical role. Deletion of *mti2* disrupts mitochondrial protein synthesis and impairs mitoribosome assembly [10]. A recent study has further demonstrated that the insertion domain of Mti2 is critical for its proper folding and for maintaining mitochondrial translation efficiency in *S. pombe* [11]. Another distinct feature of mitochondrial translation is the requirement for translational activators, which function in conjunction with the initiation factors. In the budding yeast *Saccharomyces cerevisiae*, activators such as Sov1, Cbp1, Pet309, and Mss51 regulate the translation of specific mitochondrial mRNAs [6]. Similarly, Ppr10 and Mpa1 are key activators required for efficient translation in *S. pombe* [12,13]. Due to the essential roles of mitochondria in cellular homeostasis, mitochondrial dysfunction is associated with various human diseases, including neurodegenerative disorders such as Parkinson’s and Alzheimer’s [1,14].

Mitochondrial dysfunction can impact nuclear transcription through retrograde (mitonuclear) signaling pathways, enabling mitochondria to communicate with the nucleus and trigger adaptive gene expression programs, a process known as the retrograde response [15,16,17]. The retrograde response enables cells to maintain cellular homeostasis by compensating for mitochondrial problems, such as impaired oxidative phosphorylation or loss of mitochondrial membrane potential. The retrograde response is conserved across yeasts, mammals, and other eukaryotes [18,19,20]. For example, in *S. cerevisiae*, chemical inhibition of ETC complexes induces a retrograde response [21], and in *S. pombe*, mutations in respiratory genes lead to similar coordinated changes in nuclear transcription [16,22].

Cells can also adapt their carbon metabolism in response to available sugars in the environment using nutrient-sensing regulatory networks. Glucose is the preferred carbon source for most cells, and many microorganisms have evolved regulatory mechanisms to suppress the utilization of alternative carbon sources when glucose is available. This process is referred to as carbon catabolite repression (CCR), enabling cells to respond to changing environmental carbon sources by adjusting the expression of genes involved in sugar transport, carbon metabolism, and cell growth [23,24,25]. In *S. pombe*, the CCR is mainly mediated by two conserved C_2_H_2_ zinc finger transcription factors, Scr1 and Rst2 [26,27,28]. Scr1 functions primarily as a transcriptional repressor under glucose-sufficient conditions, and its activity requires the corepressors Tup11 and Tup12 to establish full repression. Scr1 represses the expression of *fbp1* in glucose-sufficient conditions. Upon glucose depletion, Scr1 is hyper-phosphorylated by the AMP-activated protein kinase (AMPK) Ssp2, leading to its export from the nucleus. This enables the transcriptional activator Rst2 to bind to the promoter of *fbp1*, thereby activating its expression [26,27,28]. ChIP-seq analysis of Scr1, Rst2, Tup11, and Tup12 suggests a model by which the transcriptional network governing carbon metabolism involves a dynamic balance between Scr1-mediated repression and Rst2-mediated activation at shared target genes [25].

Here, we investigate whether and how the inhibition of mitochondrial translation affects the nuclear transcriptome of *S. pombe* in different carbon sources and growth phases. Proliferating *S. pombe* cells will grow mainly by fermentation in standard glucose medium, and once glucose becomes limiting, they will enter a non-dividing state (stationary phase) when mitochondrial respiration increases. Stationary phase and other non-dividing cell states have been much less studied than cell proliferation. We find that only during the stationary phase in glucose medium does inhibition of mitochondrial translation trigger an extensive transcriptional reprogramming of carbon metabolism, along with a general stress-like transcriptional response. We show that Scr1 and Rst2 contribute to this adaptive reprogramming process by regulating the expression of a shared set of genes, many of which are involved in carbon metabolism. In stationary-phase cells, Rst2 functions as a primary transcriptional activator and remains in the nucleus only when mitochondrial translation is inhibited. The retrograde response and CCR have been studied in isolation in *S. pombe*. Our findings reveal new aspects of the transcriptional response to mitochondrial defects: upon inhibition of mitochondrial translation, stationary-phase cells launch a gene expression program that combines a retrograde-like response with a response controlled by CCR regulators.

## 2. Materials and Methods

### 2.1. Fission Yeast Strains and Growth Media

*S. pombe* strains used in this study are listed in Supplementary Table S1. The deletion strains Δ*rst2* and Δ*scr1* were generated by homologous recombination using pFA6a-kanMX6 [29] in the wild-type strain *972 h^−^* background. The *S. pombe* Bioneer haploid deletion library v5.0, containing 3420 deletion mutants, was obtained from the Bähler lab stock [30,31]. The query strains (*h^+^ rst2*:: *natMX6*) for SGA with a nourseothricin cassette were constructed in a *975 h^+^* background by overlapping PCR [32]. As a control query strain for SGA, we used a wild-type strain (*h^+^ natMX6@*(*NC_003421.2:1959559_1959560ins*)), with genotype named according to [33], in which a nourseothricin cassette was inserted, hereafter referred to as Nat^R^ wild type. This insertion does not affect the fitness of the wild-type strain (Supplementary Figure S5). All deletion strains were verified by colony PCR using check primers, with all primers used in this study listed in Supplementary Table S2. Strains expressing Rst2 C-terminal-tagged GFP from its endogenous promoter were generated by overlapping PCR, as described [32]. The GFP-tagged strain does not affect cell growth and mitochondrial function (Supplementary Figure S6).

*S. pombe* cells were cultured in rich yeast extract and supplements medium (YES: 0.5% yeast extract, 225 mg/L adenine, histidine, leucine, uracil, and lysine hydrochloride) supplemented with 3% glucose for fermentative growth or 3% glycerol and 0.1% glucose for respiratory growth. Malt extract agar (MEA) media was obtained from Formedium (PCM 0810).

### 2.2. RNA-Sequencing Experiments

**Figure 1:**
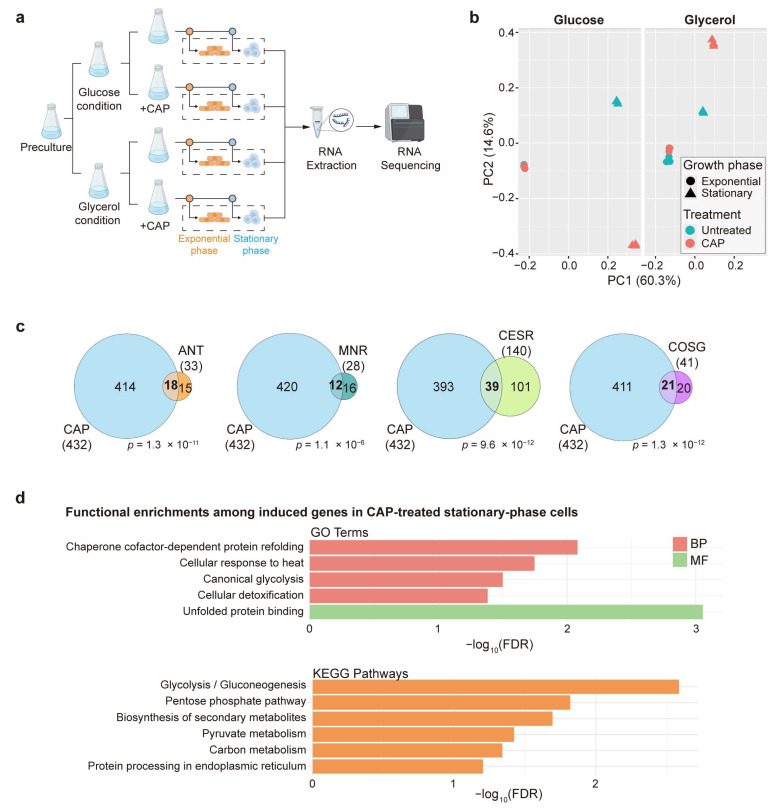
Mitochondrial translation inhibition primarily affects the transcriptome during the stationary phase. (**a**) Experimental design for RNA-seq samples used in this study. For experimental details, see the Materials and Methods Section. Three independent biological replicates were prepared for each condition. (**b**) Principal component analysis (PCA) based on rlog-transformed normalized expression values of transcriptome responses to CAP treatment under different carbon sources and growth phases. Each dot represents one biological replicate. Dot shapes indicate the growth phases (circle: exponential; triangle: stationary), and dot colors indicate CAP treatment (turquoise: untreated; pink: treated). The separate panels for glucose and glycerol conditions are derived from the same PCA using all samples and thus share the same coordinate axes. PC1 and PC2 account for 60.3% and 14.6% of the total variance, respectively. (**c**) Overlap analysis of CAP-induced genes with gene sets induced in response to other stress conditions. Statistical significance of the overlaps was calculated using Fisher’s exact test with the GeneOverlap R package (version 1.40.0) [39], with the resulting *p*-values of the overlaps indicated. The background gene set used for the analysis corresponds to all genes detected in the RNA-seq dataset in this study. (**d**) GO term and KEGG pathway enrichment analysis of 432 induced genes following CAP treatment during the stationary phase in glucose medium. GO terms are grouped by category: BP (biological process) and MF (molecular function), and were selected for non-redundancy, specificity, and significance.

The schematic overview of samples for total RNA extraction following CAP treatment is shown in Figure 1a. Briefly, cells were precultured overnight in YES medium containing 3% glucose. The cultures were subsequently harvested, washed with ddH_2_O, and diluted with fresh YES containing 3% glucose (glucose medium, fermentative condition) or 3% glycerol (glycerol medium, respiratory condition) and 0.1% glucose to an initial OD_600_ of 0.2 (Time 0). Chloramphenicol (CAP) was then added to a final concentration of 2 mg/mL, where indicated. To control for any solvent effects, 2% ethanol (EtOH) was added to untreated samples, as CAP was dissolved in EtOH. Cell growth was monitored by measuring OD_600_ every 2 h up to 18 h and every 6 h thereafter (Supplementary Figure S1b). For the preparation of the Δ*rst2* and wild-type (wt) samples, cells were precultured overnight in YES medium, diluted to an initial OD_600_ of 0.2 (Time 0), and then harvested for RNA extraction at 6 h (exponential phase) and 30 h (stationary phase). Three independent biological repeats were prepared for each condition.

Total RNA was extracted using the PureLink RNA Mini Kit (Thermo Fisher Scientific, Waltham, MA, USA) following the manufacturer’s instructions. Cells were homogenized with 0.5 mm acid-washed beads (Sigma-Aldrich, St. Louis, MO, USA) using a FastPrep instrument (MP Biomedicals, Santa Ana, CA, USA). RNA was purified with RNA Mini Columns (Qiagen, Hilden, Germany) and digested with DNase I for 15 min at room temperature to remove genomic contamination. RNA quality was assessed using the High-Sensitivity D5000 ScreenTape Assay on the TapeStation System (Agilent Technologies, Santa Clara, CA, USA). Strand-specific RNA-seq libraries were prepared using poly(A) mRNA enrichment from total RNA samples and sequenced on an Illumina platform with 150 bp paired-end reads in two lanes. Raw FASTQ files were provided for downstream analysis. Sequencing quality was assessed by FastQC (version 0.12.1, https://www.bioinformatics.babraham.ac.uk/projects/fastqc/, accessed on 29 July 2024). Transcript abundance was estimated by quasi-mapping to *S. pombe* reference genome sequences and quantified with Salmon (https://combine-lab.github.io/salmon/, accessed on 29 July 2024) [34]. Differential expression analysis was performed by DESeq2 (version 1.44.0). A linear model was constructed incorporating different carbon sources (glucose vs. glycerol), mitochondrial translation status (untreated vs. CAP treated), and growth phases (exponential vs. stationary) as independent factors. The designed model formula used for DESeq2 was specified as “~ carbon source * mitochondrial translation status * growth phase”. The *S. pombe* genome sequence and gene annotation files were obtained from PomBase [35]. Gene ontology (GO) terms and KEGG pathway enrichment analysis were performed by DAVID [36,37] with default parameters and visualized by R (version 4.4.1) [38]. Overlap analysis used in this study was performed using two approaches. Pairwise overlaps between two gene sets were assessed by the GeneOverlap R package (version 1.40.0) [39]. For overlaps among three gene sets, we used the SuperExactTest R package (version 1.1.0) [40].

### 2.3. Quantitative Cell Growth Assays in Liquid Culture

Quantitative growth assays were performed using 48-well FlowerPlates in a BioLector microbioreactor (m2p-labs GmbH, Baesweiler, Germany), as previously described [31]. Briefly, wild-type, Δ*rst2*, and Δ*scr1* strains were precultured overnight in YES medium and subsequently diluted to an initial OD_600_ of 0.2 in fresh YES. After ~4 h of cultivation to mid-exponential phase, the cultures were further diluted with 1.5 mL of fresh YES media supplemented with either 3% glucose or 3% glycerol and 0.1% glucose to an initial OD_600_ of 0.02 (Time 0). Mitochondrial translation was inhibited with the treatment of CAP at a final concentration of 2 mg/mL. Cultures were incubated in three replicates at 32 °C with shaking at 1000 rpm and 85% humidity. Growth was monitored in real time, and growth data were normalized to the initial time point (Time 0). The mean growth curves were fitted, and growth rates and lag period were calculated by grofit (version 1.1.1.1) [41]. All pairwise comparisons were analyzed using one-way ANOVA, followed by Tukey’s honest significant difference test. Data visualization was performed in R (version 4.4.1) [38].

### 2.4. Spot Assays

The wild-type, Δ*rst2*, and Δ*scr1* strains were precultured overnight in YES medium at 32 °C, and cultures were subsequently diluted to an initial OD_600_ of 0.2. CAP (dissolved in EtOH) was added to a final concentration of 2 mg/mL to inhibit mitochondrial translation, and 2% EtOH was added as a control for solvent effects. A total of 3 OD_600_ of cells were collected at 6, 12, 24, 36, and 48 h, and 10-fold serial dilutions were prepared and spotted onto YES plates. Plates were incubated at 32 °C for 3 days before being photographed.

The spot assays for wild-type strains in the SGA analysis were performed as follows. The wild-type, Δ*ade6* (see Section 2.6), and Natᴿ wild-type strains were precultured overnight in YES medium at 32 °C. Cultures were diluted to an initial OD_600_ of 0.1 and grown for 4 h to ensure collection during the logarithmic growth phase. The OD_600_ was then adjusted to 0.2, and serial dilutions were prepared and spotted onto YES plates, either without additives (control) or supplemented with the indicated stress agents: oxidative stress (0.075% MMS), heat stress (38 °C), low glucose (0.1%), non-fermentable carbon source (3% glycerol + 0.05% glucose), ER stress (75 fg/mL tunicamycin), TOR inhibition (5 µM Torin), and rapamycin + caffeine (100 µg/mL + 10 mM). Plates were incubated at 32 °C for 3 days before scanning (Supplementary Figure S5).

### 2.5. Chronological Lifespan (CLS) Assays

CLS assays assess the viability of non-dividing cells during aging by measuring colony-forming units (CFUs) at different time points. CLS assays were performed as previously described [42]. Briefly, cells were precultured overnight in YES medium at 32 °C, diluted to an initial OD_600_ of 0.02, and cultured to stationary phase (Day 0). CFUs were measured daily and normalized to the CFUs at Day 0 (100% cell viability). At each time point, aliquots of aging cultures were collected, 3-fold serially diluted, and spotted onto YES plates using a RoToR HDA pinning robot (Singer Instruments, Somerset, UK). Three independent biological replicates were prepared for each condition. Plates were incubated at 32 °C for 3 days and subsequently imaged. The scanned images were analyzed using the R package DeadOrAlive (version 1.0.0, https://github.com/JohnTownsend92/DeadOrAlive, accessed on 20 January 2025) [42].

### 2.6. Synthetic Genetic Array (SGA) Analysis

A genome-wide genetic interaction analysis was performed to identify the genes that genetically interact with *rst2*. The SGAs were carried out as previously described [43]. The deletion strain Δ*rst2* (*h^+^ rst2*:: *natMX6*) was used as a query strain to mate with the *S. pombe* Bioneer haploid deletion library v5.0, consisting of 3420 deletion mutants marked with *kanMX6*. Traditionally, the Δ*ade6* strain was used as a control strain, but we observed that this strain features some traits different from wild type, likely altering the growth and chronological lifespan (Supplementary Figure S5a,b). We, therefore, generated a new prototrophic control strain, Nat^R^ wild type (as described in Section 2.1), which shows the same fitness as the regular wild-type strain (Supplementary Figure S5c,d). The query strains were mated with the library mutant strains to create double-mutant strains on MEA plates. After incubation for 3 days at 25 °C for mating and sporulation and 3 days at 42 °C to eliminate the parental cells, the resulting colonies were selected on YES agar plates containing G418 and nourseothricin. Once colonies were sufficiently grown, they were printed onto either YES or YES containing 2 mg/mL CAP agar plates, followed by incubation at 32 °C for 3 days. The plates were imaged by an EPSON V800 scanner. Each condition was performed in three biological repeats.

The SGA image analysis was conducted in R (version 4.4.1) [38]. The colony sizes were quantified by the R package gitter (version 1.1.4) [44]. Systematic gene IDs were assigned to colony data according to the Bioneer library plate number and row/column position. Small (<50 pixels) and absent colonies were excluded to minimize false positives potentially caused by technical issues, such as an incomplete library or poorly pinned mutants. The colony sizes were subsequently normalized to reduce spatial and plate-to-plate variation effects by median smoothing and row–column median normalization [45]. Genes located within 250 kb of the query loci were excluded to avoid effects from linked loci. Genetic interaction scores (GISs) were calculated as the log_2_-transformed ratio between the normalized colony sizes of the mutant strain and the wild-type control. Genetic interaction hits were defined as genes with a GIS beyond the threshold of ±0.15 and a *p*-value less than 0.1.

### 2.7. Fluorescence Microscopy

Overnight cultures of cells expressing Rst2-GFP were diluted to an initial OD_600_ of 0.2 and grown for the indicated hours (6, 12, 18, 24, 30, and 36 h) before analyzing by fluorescence microscopy. The green fluorescence signals were captured using a Zeiss Axio Imager 2 microscope (Carl Zeiss, Jena, Germany) with excitation wavelengths of 488 nm.

## 3. Results

### 3.1. Mitochondrial Translation Inhibition Affects Genome Regulation Mainly During Stationary Phase

To investigate the effect of mitochondrial translation inhibition on the transcriptome in *S. pombe*, we performed RNA-sequencing before and after treatment inhibiting mitochondrial translation with chloramphenicol (CAP) [46,47]. We sequenced the transcriptomes of cells grown in glucose and glycerol media, both in the exponential and stationary phases. Exponentially growing cells require low mitochondrial respiratory activity in glucose media (fermentative condition), whereas they require high mitochondrial activity in glycerol media (respiratory condition). Stationary-phase cells have stopped proliferating due to carbon source depletion and require higher mitochondrial respiratory activity than exponentially growing cells in glucose media. The experimental design for the eight RNA-seq conditions is illustrated in Figure 1a. We carried out three independent biological repeats for each condition. The numbers of differentially expressed genes following CAP treatment under each condition are summarized in Table 1. The stationary-phase cells in glucose medium exhibited the strongest transcriptome response to CAP. The annotated gene lists and transcriptome-wide expression data are provided in Supplementary Dataset S1. Principal component analysis (PCA) based on regularized log (rlog)-transformed normalized expression values of the transcriptome signatures in all conditions revealed that the largest variance in the data is attributed to differences between exponentially growing and stationary-phase cells, irrespective of CAP treatment, while the second-largest variance is based on differences between CAP-treated and untreated cells in stationary-phase cells, particularly in glucose medium (Figure 1b and Table 1). In exponentially growing cells, on the other hand, CAP treatment had only a subtle effect on the transcriptome, especially in glucose medium (Figure 1b and Table 1). These results are consistent with the much higher requirement for mitochondrial respiration in stationary-phase cells, particularly in glucose medium. However, with glycerol as the carbon source, high mitochondrial respiration is required even in exponentially growing cells to sustain energy production [48]. Thus, the altered expression of only 23 genes in response to CAP treatment in this condition is surprising. Nevertheless, cell growth was almost completely abolished in CAP-treated cells in this condition (Supplementary Figure S1a), indicating that mitochondrial translation is essential for proliferation in glycerol medium, as expected.

Unlike in exponentially growing cells in glycerol medium, CAP treatment triggered a strong response in stationary-phase cells, with 391 genes showing altered expression (Table 1). Gene ontology (GO) and KEGG pathway analysis revealed that the induced genes were enriched in glycolysis/gluconeogenesis as well as genome maintenance processes, including DNA integration, recombination, and polymerase activity, while the repressed genes were enriched in nucleotide metabolism-related pathways and chromatin functions (Supplementary Figure S2). These results suggest a potential trade-off between energy metabolism and genome maintenance under mitochondrial translation stress.

CAP-treated stationary-phase cells grown in glucose medium showed the strongest transcriptome response, with 888 genes showing altered expression levels (Table 1). Therefore, the remainder of the results focus on this condition.

### 3.2. Mitochondrial Translation Inhibition During Stationary Phase Induces Genes Involved in the General Stress and Retrograde Responses

We compared the 432 genes that were induced in CAP-treated stationary-phase cells grown in glucose medium with the following gene sets induced under other stressful conditions: (1) response to treatment with antimycin A (ANT), an inhibitor of the mitochondrial ETC complex III [16]; (2) mitonuclear retrograde (MNR) response, comprising genes altered in response to ANT as well as in two respiratory-deficient mutants [16]; (3) the core environmental stress response (CESR), which includes genes responsive to oxidative stress, heavy metal stress, heat shock, osmotic stress, and DNA damage [49]; (4) the core oxidative stress genes (COSGs), which are induced in three different oxidants [50]. We observed significant overlaps between our set of induced 432 genes and all 4 stress-related gene sets induced in different conditions (Figure 1c). Various numbers of CAP-induced genes overlapped with different combinations of the other stress-related lists (Supplementary Figure S3). In contrast, the genes induced in the other CAP-treated conditions did not show significant overlaps with genes induced in any of the four stress conditions, and no significant overlaps were observed among the genes repressed in the CAP-treated conditions.

To further elucidate the biological processes associated with the genes induced in CAP-treated stationary-phase cells in glucose medium, we performed gene ontology (GO) and KEGG pathway enrichment analyses. The 432 genes were significantly enriched in terms related to stress response and protein homeostasis processes (Figure 1d). These enrichments are consistent with the significant overlaps with the responses to other stressful conditions (Figure 1c). Together, these findings indicate that mitochondrial translation inhibition triggers a transcriptional response similar to that observed in other stressful conditions, but only in stationary-phase cells grown in glucose medium.

### 3.3. Mitochondrial Translation Inhibition During Stationary Phase Induces Carbon Metabolism Genes Functioning in Non-Mitochondrial Pathways

**Figure 2:**
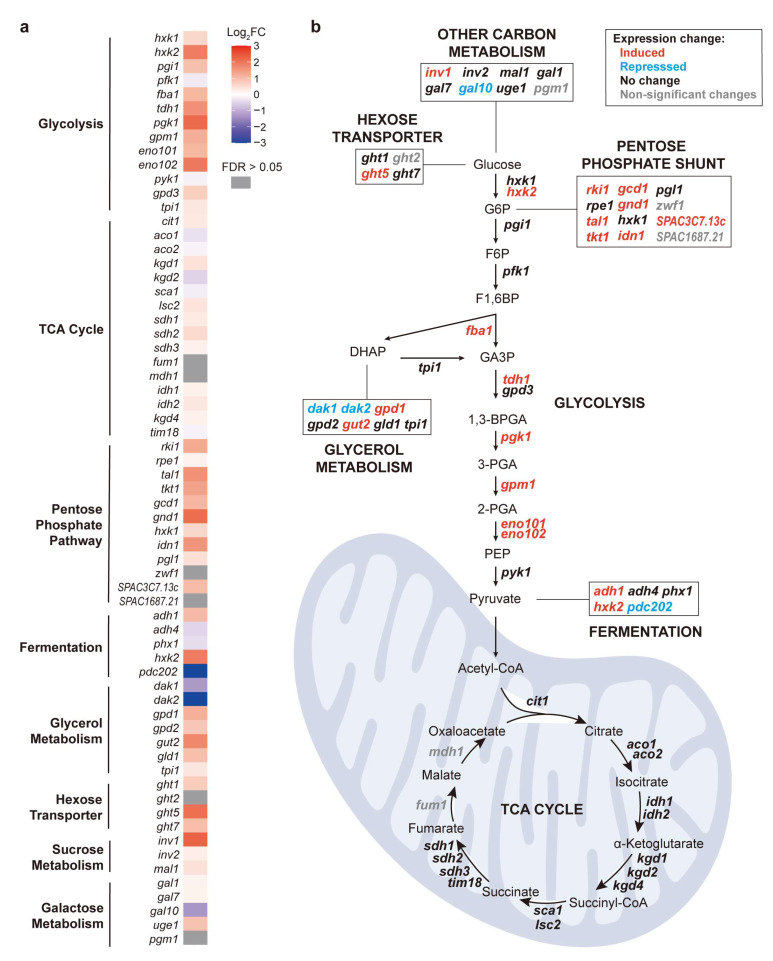
Expression changes of carbon metabolism genes in response to CAP treatment of stationary-phase cells in glucose medium. (**a**) Heatmap of expression fold changes (FCs) for genes involved in major carbon metabolism pathways. The log_2_FC values are color-coded as indicated, with grey boxes indicating non-significant expression changes (FDR > 0.05). (**b**) Carbon metabolism network scheme to map gene expression changes in response to CAP treatment in stationary-phase cells grown in glucose medium. Genes are colored according to their log_2_FC shown in (a): red indicates significantly induced genes (log_2_FC > 1), blue indicates significantly repressed genes (log_2_FC < −1), black indicates small fold changes (−1 < log_2_FC < 1), and grey indicates non-significant changes (FDR > 0.05).

When glucose is used as a carbon source (fermentative condition), exponentially growing cells primarily rely on cytosolic glycolysis to convert glucose to pyruvate, which is then fermented to ethanol [51]. During the stationary phase, however, cells shift to mitochondrial respiration, whereby pyruvate is oxidized through the tricarboxylic acid (TCA) cycle and oxidative phosphorylation to generate ATP [52]. Figure 1d shows that the genes induced in CAP-treated stationary-phase cells grown in glucose medium were also significantly enriched in several pathways related to carbon metabolism, including glycolysis, the pentose phosphate pathway, and pyruvate metabolism (Figure 1d). In contrast, no significant enrichments were detected among the genes repressed in response to CAP treatment. To further characterize this response, we examined all genes associated with major carbon metabolic pathways in PomBase [53], including glycolysis, the TCA cycle, the pentose phosphate pathway, fermentation, and glycerol metabolism. Figure 2a shows the expression changes of all these genes in CAP-treated stationary-phase cells grown in glucose medium, grouped by the different metabolic pathways. Notably, the pathways that include most of the induced genes all function in the cytoplasm, outside of mitochondria and upstream of the TCA cycle, most notably genes functioning in glycolysis and the pentose phosphate shunt (Figure 2b). Together, these findings indicate that the inhibition of mitochondrial translation activates a substantial metabolic reprogramming that enhances the cytosolic carbon flux, likely as a compensatory mechanism shifting energy metabolism from respiration to fermentation to maintain cellular homeostasis when mitochondrial oxidative phosphorylation is impaired.

### 3.4. The Scr1 and Rst2 Transcription Factors Regulate Common Metabolic Genes upon Inhibition of Mitochondrial Translation in Stationary-Phase Cells

**Figure 3:**
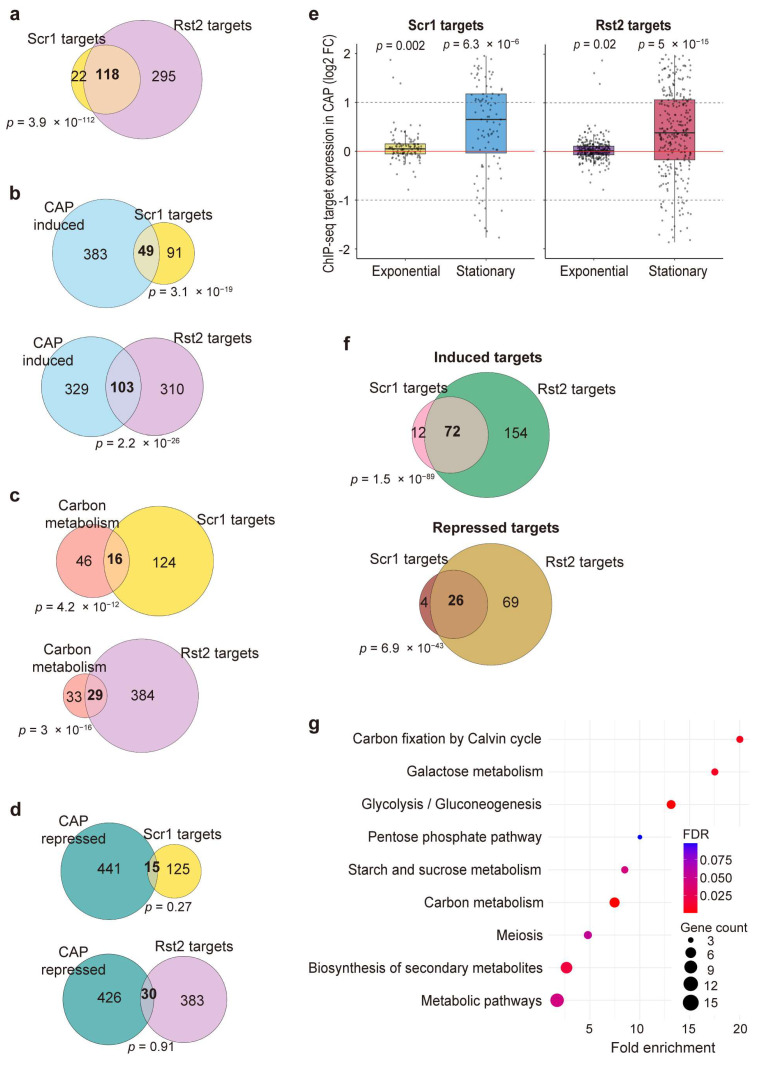
Scr1 and Rst2 regulate shared metabolic genes in response to CAP treatment. (**a**) Venn diagram to show the overlap between published Scr1 and Rst2 target genes [25]. Statistical significance of overlaps was calculated using Fisher’s exact test with the GeneOverlap R package (version 1.40.0) [39], with the resulting *p*-values of the overlaps indicated. The background gene set used for the analysis corresponds to all genes detected in the RNA-seq dataset in this study. (**b**–**d**) Overlap analysis of CAP-induced genes (**b**), carbon metabolism genes in Figure 2 (**c**), and CAP-repressed genes (**d**) with Scr1 and Rst2 target genes, with the resulting *p*-values of the overlaps indicated. Statistical significance of overlaps was calculated using Fisher’s exact test with the GeneOverlap R package (version 1.40.0) [39], with the resulting *p*-values of the overlaps indicated. The background gene set used for the analysis encompasses all genes detected in the RNA-seq dataset in this study. (**e**) Transcriptional response of Scr1 and Rst2 target genes to CAP treatment. Box plots show log_2_ fold changes in gene expression of Scr1 and Rst2 targets in CAP-treated versus untreated cells during exponential and stationary phases, as indicated. Substantial changes in gene expression (mainly induction) of Scr1 and Rst2 targets were observed predominantly during the stationary phase. Red horizontal lines mark log_2_ fold change = 0 (no change), while dashed lines mark log_2_ fold-change thresholds of ±1. The *p*-values were calculated using the Wilcoxon signed-rank test [54]. (**f**) Overlap analysis of Scr1 and Rst2 targets that were induced (top) or repressed (bottom) based on the RNA-seq data shown in (e), with the *p*-values of the overlap indicated. (**g**) KEGG pathway enrichment analysis of Scr1 and Rst2 target genes that are induced in our data. The most enriched pathways are related to carbon metabolism. Dot size represents gene count per pathway, and dot color indicates FDR values, as shown on the right. The analysis was performed using DAVID [36,37] with default parameters and visualized by R (version 4.4.1, R Foundation for Statistical Computing, Vienna) [38].

The CAP-induced gene expression response appears to be a complex program that likely involves multiple regulatory factors. The Scr1 and Rst2 transcription factors are key regulators of the CCR (see the Introduction Section). ChIP-seq experiments have identified 140 Scr1 and 413 Rst2 target genes in glucose and glycerol media, respectively, where these factors are most active [25]. As expected, these two gene sets share many common target genes (Figure 3a). To investigate the involvement of Scr1 and Rst2 in the CAP-induced gene expression response, we performed an overlap analysis between their published target genes and our 432 genes induced in CAP-treated stationary-phase cells grown in glucose medium. These genes showed significant overlaps with both the Scr1 and Rst2 target genes (Figure 3b). Moreover, many of the carbon metabolism genes analyzed above (Figure 2) were included among the Scr1 and Rst2 target genes (Figure 3c). In contrast, the 456 repressed genes in CAP-treated stationary-phase cells showed no significant overlaps with Scr1 or Rst2 target genes (Figure 3d).

We then tested whether the Scr1 and Rst2 target genes showed altered expression in response to CAP treatment in exponentially growing or stationary-phase cells in glucose medium. On average, both the Scr1 and Rst2 target genes were significantly induced in stationary-phase cells, while they showed only minor expression differences in exponentially growing cells (Figure 3e). These findings further support the conclusion that Scr1 and Rst2 play an important role in regulating the strong transcriptional response to mitochondrial translation inhibition in stationary-phase cells.

To dissect the regulatory relationship between Scr1 and Rst2, we looked for their target genes that were significantly induced or repressed in our RNA-seq data of CAP-treated stationary-phase cells. Notably, the target genes common to Scr1 and Rst2 included significant numbers of both induced and repressed genes, although fewer of the latter (Figure 3f). In contrast, no overlap was observed between induced Scr1 target genes and repressed Rst2 target genes, or vice versa. These results indicate that Scr1 and Rst2 cooperatively regulate a set of common genes, and most of these genes are induced in response to CAP treatment. KEGG pathway analysis of the genes that are positively regulated by both Scr1 and Rst2 revealed significant enrichments in several processes related to carbon metabolism (Figure 3g). Collectively, these findings support a model of functional convergence, in which Scr1 and Rst2 jointly promote the reprogramming of carbon metabolism in response to mitochondrial translation inhibition.

### 3.5. Scr1 and Rst2 Antagonistically Affect Cellular Growth and Viability upon Inhibition of Mitochondrial Translation

**Figure 4:**
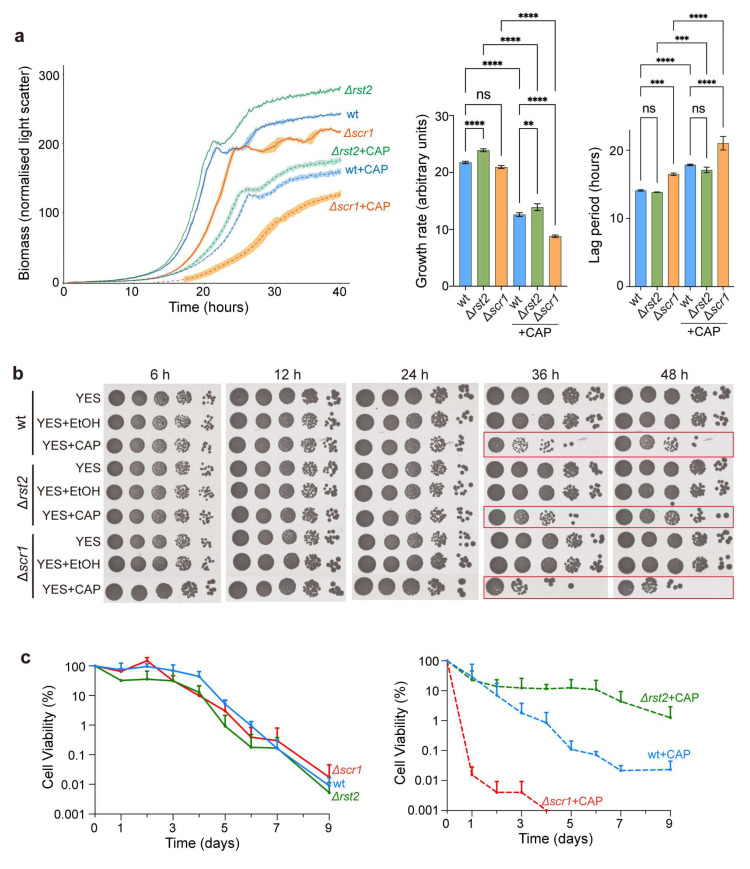
Scr1 and Rst2 affect growth and viability of CAP-treated cells. (**a**) Growth measurement of wild-type (wt), ∆*rst2*, and ∆*scr1* strains cultured in YES medium with or without CAP treatment. Left graph: growth curves were measured using a microbioreactor, as described [31]. Mean growth curves (biomass accumulation) are shown, along with the standard deviation (shaded regions) for three independent replicates. Growth rates and lag phases (middle and right panels) were quantified from the left graph using the R package grofit (version 1.1.1.1) [41]. The statistical analysis was conducted by one-way ANOVA, followed by Tukey’s honest significance test (ns, not significant; **, *p* < 0.01; ***, *p* < 0.001; ****, *p* < 0.0001). (**b**) Viability assays of wild-type, ∆*rst2*, and ∆*scr1* strains with and without CAP treatment, with 2% EtOH serving as a solvent control. Cells grown to an initial OD_600_ in the media indicated on the left were collected after the indicated time points, and 10-fold serial dilutions were spotted onto YES solid plates. The plates were photographed after 3 days. The red boxes highlight markedly reduced viability at 36 h and 48 h. (**c**) Chronological lifespan assays of wt, ∆*rst2*, and ∆*scr1* strains without (left graph) or with (right graph) CAP treatment. Cells were grown in YES medium to stationary phase (Day 0). A proxy for cell viability was determined over time by a robotics-based colony-forming unit (CFU) assay [42], with values normalized to Day 0 (100% viability). CFUs were measured daily from Day 0 to Day 7 and at Day 9. At each time point, aliquots of aging cultures were collected, 3-fold serially diluted, and spotted onto YES plates, which were imaged after three days. Three independent biological replicates were prepared for each condition. Error bars represent standard deviation (SD) and are shown only in the upward direction for clarity on a log-scale *Y* axis.

To further investigate the roles of Rst2 and Scr1 in CAP-treated cells, we constructed Δ*rst2* and Δ*scr1* deletion strains. We then assessed various traits of the cells lacking Rst2 or Scr1 as a function of CAP treatment. Without CAP treatment, Δ*rst2* cells showed a slightly faster growth rate, shorter lag phase, and higher final cell mass compared to wild-type cells, while Δ*scr1* cells showed a slower growth rate, prolonged lag phase, and lower final cell mass (Figure 4a). With CAP treatment, all three strains exhibited reduced overall growth patterns, but with the same relative differences of increased growth patterns in Δ*rst2* and decreased growth patterns in Δ*scr1* compared to wild-type cells (Figure 4a). To assess whether CAP affects cell viability over time, we performed serial-dilution spot assays at multiple time points following CAP treatment. In untreated cells, deletion of either *rst2* or *scr1* did not significantly affect cell viability at any time points compared to wild type (Figure 4b). CAP-treated cells of all three strains, on the other hand, showed markedly reduced viability at later times of culturing (36 h and 48 h), with ∆*rst2* showing slightly higher viability and ∆*scr1* slightly lower viability compared to wild-type cells (Figure 4b). This result suggests differences in the chronological lifespan (CLS) of stationary-phase cells between the three strains. To test this prediction, we conducted CLS assays to measure the longer-term viability of stationary-phase cells. Without CAP treatment, all strains showed similar CLS profiles over time. With CAP treatment, however, ∆*rst2* cells showed a substantially extended CLS compared to wild-type cells, maintaining some viability up to Day 9, whereas ∆*scr1* cells showed a dramatically shortened CLS, with a sharp decrease of viability already by Day 1 (Figure 4c and Figure S4).

To explore whether the opposing effects of Scr1 and Rst2 on the CLS could be attributed to distinct target genes, we compared the transcriptional profiles of ∆*scr1* [25] and ∆*rst2* (this study) with previously identified long- and short-lived mutants, in which gene deletions extend or shorten the CLS, respectively [42]. Among the genes repressed in ∆*scr1* but not in ∆*rst2* cells, two deletion mutants (*abp2* and *cnp3*) have been reported to shorten the CLS [42]. Notably, *abp2* showed a 2.7-fold reduction after CAP treatment. Conversely, among the genes repressed in ∆*rst2* cells but not repressed (or not detected) in ∆*scr1* cells, three deletion mutants (*cyp4*, *mug27*, and *gal1*) have been reported to extend the CLS [42]. These distinct gene expression patterns might explain the opposite effects of Scr1 and Rst2 on the CLS under conditions of mitochondrial translation inhibition. Collectively, these findings indicate that Scr1 and Rst2 play antagonistic roles in the cellular adaptation to mitochondrial translation inhibition during the stationary phase.

### 3.6. Rst2 Activates Carbon Metabolism Genes in Stationary-Phase Cells upon Inhibition of Mitochondrial Translation

**Figure 5:**
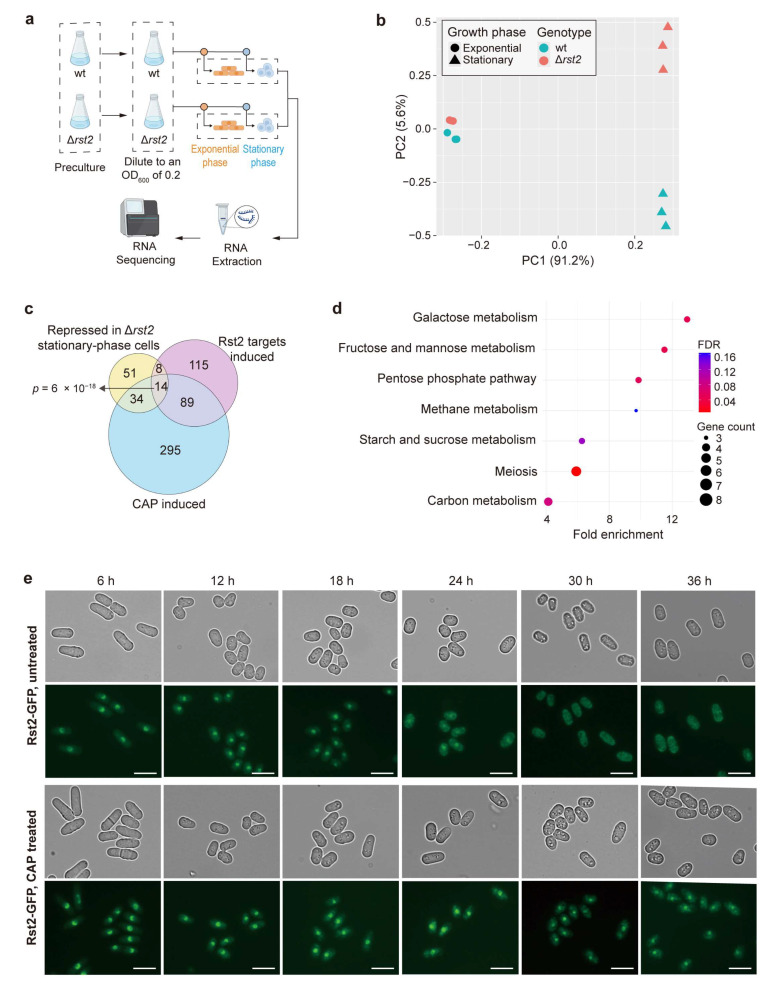
Rst2 contribution to gene regulation in response to mitochondrial translation inhibition during the stationary phase. (**a**) Experimental design of wild-type (wt) and Δ*rst2* strains used for RNA-seq analysis. For experimental details, see the Materials and Methods Section. Three independent biological replicates were prepared for each condition. (**b**) PCA based on rlog-transformed normalized expression values of transcriptome responses from ∆*rst2* and wild-type (wt) cells collected during exponential and stationary phases. Each dot represents one biological replicate. Dot shapes indicate the growth phases (circle: exponential; triangle: stationary), and dot colors indicate the genotype (turquoise: wt; pink: ∆*rst2*). PC1 and PC2 account for 91.2% and 5.6% of the total variance, respectively. (**c**) Overlap analysis of genes repressed in Δ*rst2* cells during the stationary phase compared to CAP-induced genes during the stationary phase and to Rst2 target genes [25]. The *p*-value represents the significance of the overlap among the three gene sets. (**d**) KEGG pathway enrichment analysis of the genes repressed in Δ*rst2* cells during the stationary phase. The most enriched pathways are related to carbon metabolism and meiosis. Dot size represents gene count per pathway, and color indicates FDR values, as shown on the right. The analysis was performed using DAVID [36,37] with default parameters and visualized by R (version 4.4.1, R Foundation for Statistical Computing, Vienna) [38]. (**e**) Subcellular localization of Rst2 in untreated and CAP-treated cells. Wild-type cells expressing Rst2-GFP under its endogenous promoter were grown in YES medium to an initial OD_600_ of 0.2 (0 h). Cells were then treated with CAP and collected at the indicated time points for fluorescence microscopy. Scale bar, 10 μm.

The gene expression changes in response to CAP treatment are most pronounced during the stationary phase, a state triggered by glucose limitation. Thus, Rst2 is expected to play a dominant regulatory role in this condition based on its known function in the absence of glucose [25]. We, therefore, analyzed the specific contribution of Rst2 to this adaptive response. To this end, we performed RNA-seq of Δ*rst2* and wild-type strains for both exponential and stationary phases (Figure 5a). PCA based on rlog-transformed normalized expression values of the transcriptome signatures in these conditions revealed that the biggest transcriptional differences were caused by differences in the growth phases (Figure 5b). In addition, deletion of *rst2* had a major impact on the transcriptome during the stationary phase, but not during the exponential phase (Figure 5b). This phase-specific transcriptome response resembles the pattern observed for CAP treatment (Figure 1b). A total of 137 genes were differentially expressed in Δ*rst2* compared to wild-type cells during the stationary phase, with 30 genes induced and 107 genes repressed. These results indicate that Rst2 primarily functions as a transcriptional activator, consistent with the analyses of Rst2 target genes described above (Figure 3e,f) and the known function of Rst2 in the CCR response [25].

We then checked whether the genes whose expression is lowered in stationary-phase Δ*rst2* cells are part of the CAP-induced response. To this end, we compared the repressed genes in Δ*rst2* stationary-phase cells with both the induced Rst2 target genes (Figure 3f) and the genes induced in CAP-treated stationary-phase cells grown in glucose medium. All three lists shared substantial numbers of genes (Figure 5c). These results indicate that Rst2 is required for the regulation of a substantial proportion of the genes induced in CAP-treated stationary-phase cells. Accordingly, KEGG pathway analysis of the repressed genes in Δ*rst2* stationary-phase cells revealed significant enrichments of carbon metabolism pathways (Figure 5d). Genes involved in meiosis were also significantly enriched (see also Figure 3g), consistent with the established role of Rst2 in activating *ste11* expression, which encodes a key transcription factor regulating sexual differentiation [55,56].

To assess whether CAP treatment affects the subcellular localization of Rst2, we tagged the genomic *rst2* gene with GFP to express a Rst2-GFP fusion protein. We then monitored the dynamics of Rst2-GFP in cells grown in glucose medium with or without CAP (Figure 5e). In untreated cells, Rst2 was predominantly localized to the nucleus during the exponential phase but gradually shifted to the cytoplasm during the stationary phase. In contrast, in CAP-treated cells, Rst2 remained localized in the nucleus during the stationary phase (Figure 5e). This nuclear retention further points to Rst2 contributing to the transcriptional response to CAP treatment on stationary-phase cells. Together, these findings establish Rst2 as an important regulator for the transcriptional reprogramming of carbon metabolism in stationary-phase cells adapting to the inhibition of mitochondrial translation.

### 3.7. Rst2 Genetically Interacts with Genes Involved in Stress Protection and Nutrient Response

To systematically uncover functional relationships of Rst2 with diverse biological processes, we generated a new Δ*rst2* deletion strain (*rst2::natMX6*), containing a marker suitable for a query mutant to assay genetic interactions with all 3420 non-essential prototrophic deletion mutants using the SGA method [57,58]. Genetic interactions were measured in glucose medium, both with and without CAP, based on colony size as a proxy for double-mutant fitness compared to the control mutant (see the Methods Section 2.6). We observed 246 negative and 145 positive genetic interactions for untreated double mutants, and 247 negative and 156 positive genetic interactions for CAP-treated double mutants (Supplementary Dataset S3). Significant overlaps of 74 positive (*p* = 6.8 × 10^−59^) and 171 negative (*p* = 4.3 × 10^−146^) interactions were identified between the untreated and CAP-treated conditions. Functional enrichment analysis using AnGeLi [59] revealed that these interacting genes in both untreated and CAP-treated conditions were significantly enriched for stress-related phenotypes, including the fission yeast phenotype ontology (FYPO) terms [42,60,61] ‘increased sensitivity to chemical’ (*p* = 5.5 × 10^−15^ and *p* = 1.0 × 10^−13^ for untreated and CAP-treated, respectively), ‘sensitive to DNA-damaging agents’ (*p* = 4.7 × 10^−7^ and *p* = 9.5 × 10^−9^), and ‘sensitive to hydrogen peroxide’ (*p* = 7.9 × 10^−4^ and *p* = 4.6 × 10^−5^). The term ‘decreased mating efficiency’ (*p* = 2.7 × 10^−4^ and *p* = 9.5 × 10^−5^) was also enriched, consistent with the enriched KEGG pathway ‘meiosis’ observed in Figure 3g and Figure 5d. Moreover, in untreated cells, the interacting genes additionally showed enrichment for several GO terms [62] related to ‘response to nutrient levels’ (*p* = 2.1 × 10^−3^) and ‘response to starvation’ (*p* = 3.7 × 10^−3^).

Among the 171 negative genetic interactions shared between untreated and CAP-treated conditions, four genes (*gal1*, *ght8*, *SPAC212.04c*, and *SPBC1861.05*) were also repressed genes in Δ*rst2* stationary-phase cells—*gal1* and *ght8* are involved in sugar metabolism and transport, supporting a role of Rst2 in maintaining glucose-responsive gene expression during mitochondrial stress. In addition, 14 genes overlapped with genes that are induced in CAP-treated stationary-phase cells, including *ppr5*, encoding a mitochondrial pentatricopeptide repeat protein that negatively regulates mitochondrial translation [63], and *rec24*, encoding a meiotic recombination protein [64,65], consistent with the enriched KEGG pathways related to meiosis (Figure 3g and Figure 5d). Collectively, our genetic interaction screen complements the transcriptomic data and highlights candidate genes that may work in parallel or together with Rst2 to promote the adaptation of stationary-phase cells to mitochondrial dysfunction.

## 4. Discussion

Mitochondrial translation is essential for cellular energy production and other mitochondrial functions [4]. Dysfunction of mitochondria is communicated to the nucleus through retrograde signaling, triggering adaptive gene expression programs [15,16]. Here, we investigated the transcriptome changes in response to impaired mitochondrial translation, induced by CAP treatment, in different metabolic and growth conditions. Our RNA-seq results indicated that CAP treatment primarily induces transcriptome changes in stationary-phase cells, whereas exponentially growing cells exhibit only a minor response (Figure 1b).

Surprisingly, in cells growing under a respiratory condition that requires high mitochondrial activity (glycerol medium), only 23 genes were differentially expressed upon inhibition of mitochondrial translation (Table 1). This limited response could indicate that the six-hour CAP treatment during exponential growth was insufficient to elicit a stronger transcriptional response. Such short-term CAP treatment is known to trigger only a modest reduction in the protein levels of mtDNA-encoded ETC subunits in the exponential phase, whereas in the stationary phase, after longer CAP treatment, the levels of these proteins are strongly repressed [47]. Nevertheless, CAP treatment in glycerol almost completely abolished cell growth (Figure S1). Thus, it is also possible that these cells are too impaired to launch a robust transcriptional response.

GO enrichment analysis of the differentially regulated genes in CAP-treated cells in the different conditions revealed that only the 432 genes induced in stationary-phase cells grown in glucose medium significantly overlapped with genes that were differentially expressed in response to various other stress conditions (Figure 1c). These findings suggest that mitochondrial translation inhibition in these stationary-phase cells triggers a response similar to other stresses, including the retrograde response. However, we note that the published stress-related gene sets used for comparison are from proliferating cells rather than stationary-phase cells. We included these datasets primarily as reference signatures to evaluate similarities between CAP-induced changes and previously characterized transcriptional programs. Furthermore, only these 432 genes were significantly enriched in processes and pathways related to carbon metabolism, particularly cytoplasmic pathways that provide alternatives to mitochondrial respiration (Figure 1d and Figure 2). These transcriptome changes are consistent with a reprogramming of energy metabolism from respiration to fermentation to cope with the mitochondrial dysfunction.

Our results indicate that the CAP-induced transcriptional reprogramming of carbon metabolism involves Scr1- and Rst2-mediated regulation (Figure 3). Scr1 primarily acts as a transcriptional repressor under glucose-sufficient conditions, while Rst2 functions as a transcriptional activator when glucose is limited [25]. Both Δ*scr1* and Δ*rst2* mutants displayed normal cell viability and lifespans under control conditions (Figure 4), consistent with published results [25,56]. However, CAP-treated Δ*scr1* cells showed a dramatically shortened CLS, while Δ*rst2* cells featured an extended CLS compared to wild-type cells (Figure 4c). These results suggest that Scr1 and Rst2 exert opposing regulatory roles in response to inhibition of mitochondrial translation. Scr1 and Rst2 regulate a common set of target genes, while also each controlling distinct sets of genes (Figure 3a). The Scr1 and Rst2 target genes were, on average, significantly induced during the stationary phase in CAP-treated cells (Figure 3e), and they were enriched in carbon metabolism processes (Figure 3g). Together with published results, these findings support a model of functional convergence, where Scr1 and Rst2 may act as a transcriptional ‘brake’ and ‘accelerator’, respectively, to tune the expression of carbon metabolism genes in response to mitochondrial defects. Similar regulators exist in *S. cerevisiae*, where Mig1 is the ortholog of Scr1 that controls carbon source utilization [66,67], while Cat8 is the ortholog of Rst2 that activates metabolic genes under glucose-limited conditions [68]. These parallels suggest that coordination between glucose repression and metabolic adaptation via CCR regulators represents a conserved strategy across divergent yeast species.

Our findings further suggest that Rst2 primarily functions as a transcriptional activator for the induction of CAP-responsive metabolic genes in stationary-phase cells, based on RNA-seq (Figure 5a–d) and genetic interaction data. Rst2 was predominantly localized to the nucleus during the exponential phase and gradually moved to the cytoplasm during the late stationary phase (Figure 5e). In CAP-treated cells, however, Rst2 was retained in the nucleus during the stationary phase. This result supports the conclusion that Rst2 plays an important role in the transcriptional response of stationary-phase cells to mitochondrial dysfunction. The Rst2 localization pattern we observed in control cells seems to contradict published findings, where Rst2 is typically cytoplasmic under glucose-rich conditions and translocates to the nucleus only upon glucose depletion, regulated via AMPK-mediated phosphorylation [69]. However, a cytoplasmic localization of Rst2 has been observed in overnight cultures [70], which corresponds to our stationary-phase condition. Moreover, Rst2 localization may not be exclusively regulated by glucose levels, as illustrated by the following examples. Mutations in the Pka1 kinase that phosphorylates Rst2 in glucose-sufficient conditions lead to nuclear accumulation of Rst2 [71], and Rst2 nucleocytoplasmic shuttling is impaired by deletion of ETC complex subunits [70]. Furthermore, these studies typically examined acute changes (minutes to hours) of Rst2 dynamics in response to glucose levels, whereas we captured Rst2 localization over longer times in steady-state conditions. Another difference is that we used rich medium for consistency with the RNA-seq conditions, while the other studies used minimal medium. Together, these results highlight the influence of broader metabolic and signaling contexts, besides glucose levels, that affect Rst2 function and localization. A recent study identified high-occupancy target (HOT) regions in the *S. pombe* genome that are bound by multiple transcription factors, and Rst2 was enriched at these HOT regions [72]. This observation might reflect a broader involvement of Rst2 in global gene regulation.

## 5. Conclusions

We investigated whether and how the inhibition of mitochondrial translation affects genome regulation in different metabolic conditions and growth phases. During the stationary phase in glucose medium, CAP treatment triggered a transcriptional reprogramming of carbon metabolism from respiration to fermentation, along with changes resembling general stress and retrograde responses. The CCR regulators Scr1 and Rst2 controlled a shared set of metabolic genes in response to CAP treatment. Scr1 and Rst2 had opposing effects on the viability of CAP-treated stationary-phase cells. In these cells, which were in a glucose-limited state, Rst2 remained localized in the nucleus and functioned as a transcriptional activator of carbon metabolism genes. Genetic interactions pointed to functional relationships of Rst2 with stress and starvation responses. Together, our findings revealed a crosstalk between stress/retrograde response-like signatures and the CCR regulators Scr1 and Rst2 and highlighted a coordinated regulatory strategy that reprograms carbon metabolism in response to mitochondrial dysfunction. These findings deepen our understanding of mitochondrial–nuclear communication and the resulting transcriptome regulation in fission yeast and may also inform how other eukaryotic cells adapt their metabolism under mitochondrial stress.

## Figures and Tables

**Table 1:** Numbers of differentially expressed protein-coding transcripts after CAP treatment in the different experimental conditions. Induced and repressed genes are defined as transcripts that show expression changes in CAP-treated cells relative to untreated control cells in the same condition, with a significance threshold of *p* < 0.05 and |log_2_(Fold Change)| > 1. A detailed list of these annotated genes is provided in Supplementary Dataset S1.

Condition	Induced	Repressed
Glucose_Exponential	9	2
Glucose_Stationary	432	456
Glycerol_Exponential	15	8
Glycerol_Stationary	141	250

## Data Availability

The RNA-seq data have been submitted to GEO under the accession number PRJNA1262735: https://www.ncbi.nlm.nih.gov/sra/PRJNA1262735 (accessed on 12 September 2025).

## Supplementary Materials

The following supporting information can be downloaded at: https://www.mdpi.com/article/10.3390/biom15101354/s1, Table S1: List of *S. pombe* strains used in this study. Table S2: Primers used in this study. Figure S1: Growth measurement with or without CAP treatment in glucose or glycerol media. Figure S2: GO terms and KEGG pathway enrichment analysis of 141 induced (a) and 250 repressed (b) genes following CAP treatment during the stationary phase in glycerol medium. Figure S3: Overlaps of CAP-induced genes with multiple stress-responsive gene sets. Figure S4: Chronological lifespan assays. Figure S5: The Natᴿ wild-type strain, unlike the Δ*ade6* strain, shows no phenotypic differences compared to the original wild-type strain. Figure S6: Growth assessment of the Rst2-GFP strain. Dataset S1: Transcriptomic changes after the inhibition of mitochondrial translation under different carbon sources and growth phases. Dataset S2: Transcriptomic changes in Δ*rst2* compared to wild-type cells. Dataset S3: SGA assay data. File S1: Original microscopy images.

## Abbreviations

The following abbreviations are used in this manuscript:

mtDNA	Mitochondrial DNA
ETC	Electron transport chain
CCR	Carbon catabolite repression
AMPK	AMP-activated protein kinase
GO	Gene ontology
ANT	Antimycin A treatment
CESR	Core environmental stress response
COSG	Core oxidative stress gene
MNR	Mitonuclear retrograde
TCA	Tricarboxylic acid
CLS	Chronological lifespan
FYPO	Fission yeast phenotype ontology
