# Supplementary material for: Mitochondrial Translation Inhibition Triggers an Rst2-Controlled Transcriptional Reprogramming of Carbon Metabolism in Stationary-Phase Cells of Fission Yeast
Source: Biomolecules. 2025 Sep 24;15(10):1354. doi: 10.3390/biom15101354 (PMC12564598; doi:10.3390/biom15101354)
Supplement: Supplementary file 1 [file biomolecules-15-01354-s001.zip › Supplementary information_clean copy.pdf]

Supplementary materials

Mitochondrial translation inhibition triggers a Rst2-controlled transcriptional reprogramming of carbon metabolism in stationary-phase cells of fission yeast

Ying Luo [1,2], Shaimaa Hassan [1], Saniya Raut [1] and Jürg Bähler [1]*

[1]Institute of Healthy Ageing, Department of Genetics, Evolution & Environment, University College London, London WC1E 6BT, United Kingdom

[2]Jiangsu Key Laboratory for Microbes and Genomics, School of Life Sciences, Nanjing Normal University, Nanjing 210023, China

* Correspondence: j.bahler@ucl.ac.uk (J.B.)

Table S1: *S. pombe* strains used in this study

Strain	Genotype	Source
JB22	*h*$^-$ 972 (*S. pombe* wild type)	Lab stock
yLY1	*h*$^-$ *rst2*:: *kanMX6*	This study
yLY2	*h*$^-$ *scr1*:: *kanMX6*	This study
yLY3	*h*$^+$ *rst2*:: *natMX6*	This study
yHS1	*h*$^+$ *natMX6@(NC_003421.2:1959559_1959560ins)*	This study

Table S2: Primers used in this study

Primer Name	Primer Sequence (5′ to 3′)
Primers for deletion of *rst2*	
Δ*rst2*-up-F	CTTGTTTATCTGTGCTATTACTG
Δ*rst2*-up-R	TATTCTGGGCCTCCATGTCGATAAAACAAGTTTTC
Δ*rst2*-kanMX6-F	ACTTGTTTTATCGACATGGAGGCCCAGAATAC
Δ*rst2*- kanMX6-R	TATAAAGATATGAAAAAAACAGTATAGCGACCAGC ATTCAC
Δ*rst2*-down-F	GTGAATGCTGGTCGCTATACTGTTTTTTTCATATCTT TATATTTTATAC
Δ*rst2*-down-R	GTGCATGCAGAGTGAATG
Primers for verification of *scr1* deletion	
Δ*scr1*-yz-up-F	TTTTCCAATCCTCTCTCATCGT
Δ*scr1*-yz-kanMX6-R	GGACAATTCAACGCGTC
Δ*scr1*-yz-kanMX6-F	GCCTGTTGAACAAGTCTG
Δ*scr1*-yz-natMX6-R	GCATGCTCATGTAGAGCGCC
Δ*scr1*-yz-natMX6-F	GTACCACTCTTGACGACACG
Δ*scr1*-yz-down-R	CTGAACAATGGAACCTCAAATG
Primers for verification of *rst2* deletion	
Δ*rst2*-yz-up-F	CCTTACATTTCCGATTCTCC
Δ*rst2*-yz-kanMX6-R	GGACAATTCAACGCGTC
Δ*rst2*-yz-kanMX6-F	GCCTGTTGAACAAGTCTG
Δ*rst2*-yz-natMX6-R	GCATGCTCATGTAGAGCGCC
Δ*rst2*-yz-natMX6-F	GTACCACTCTTGACGACACG
Δ*rst2*-yz-down-R	TGTTCTTTTAAGGGCCGTC

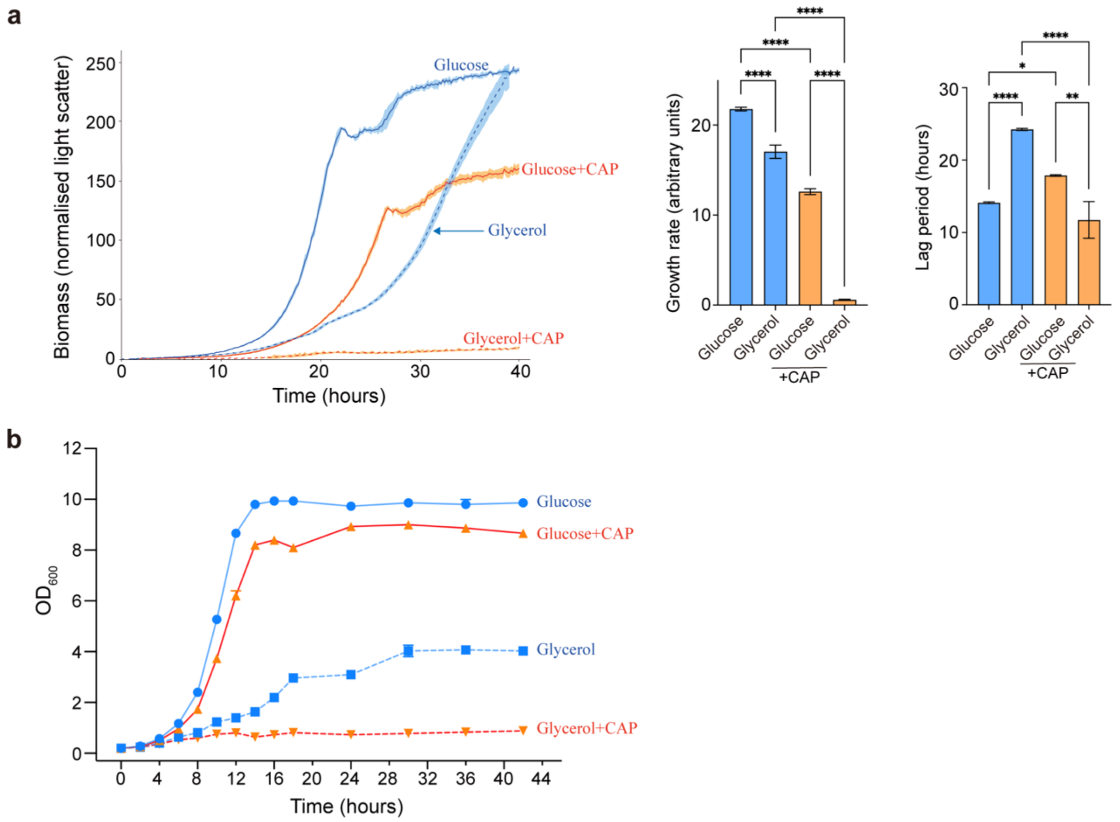

Fig. S1 Growth measurement with or without CAP treatment in glucose or glycerol media. (**a**) Quantitative growth assays of wild-type cells with or without CAP treatment in glucose or glycerol media. Cells were precultured overnight in YES medium and subsequently diluted to an initial OD_{600} of 0.2 in fresh YES. After ~4 hours of cultivation to mid-exponential phase, the cultures were further diluted with 1.5 mL of fresh YES media supplemented with YES containing either 3% glucose (Glucose) or 3% glycerol and 0.1% glucose (Glycerol) to an initial OD_{600} of 0.02 (Time 0). Growth (biomass accumulation) was monitored in real time using a microbioreactor, with measurements taken every 10 min and normalised to Time 0. Left graph: Mean growth curves are shown with standard deviation (shaded regions) from three independent replicates. Middle and right graphs: Growth rates and lag phases were quantified from the left graph. The statistical analysis was conducted by one-way ANOVA, followed by Tukey's honest significance test (*, p <0.05; **, p <0.01; and ****, p <0.0001). (**b**) Growth curves (OD_{600}) of cultures grown under the same conditions as those used for RNA extraction. Cells were precultured overnight in YES medium, and cultures were diluted to an initial OD_{600} of 0.2 (Time 0) as described in Methods 2.2. OD_{600} was measured every 2 hours up to 18 hours and every 6 hours thereafter. Growth curves are representative of three independent experiments.

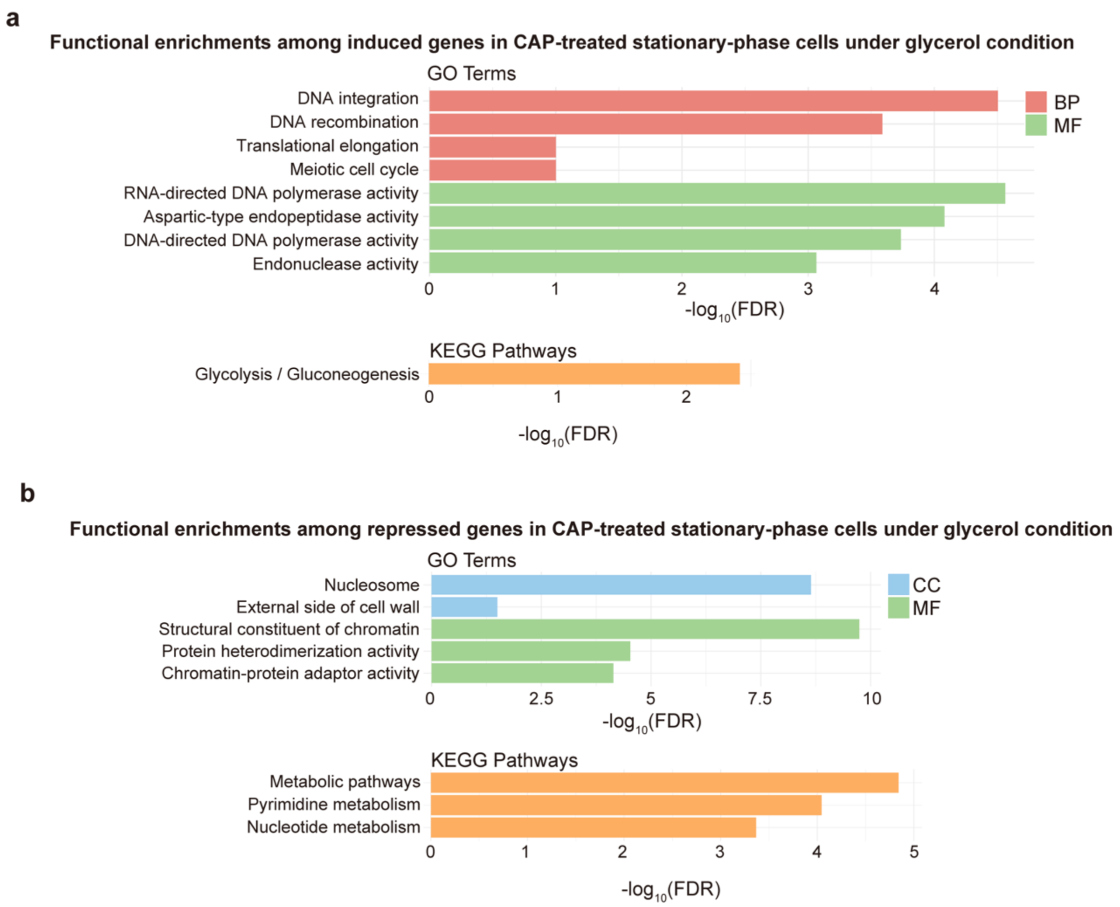

Fig. S2 GO term and KEGG pathway enrichment analysis of 141 induced (a) and 250 repressed (b) genes following CAP treatment during stationary phase in glycerol medium. GO terms are grouped by category: BP (Biological Process), CC (Cellular Component), and MF (Molecular Function), and were selected for non-redundancy, specificity, and significance.

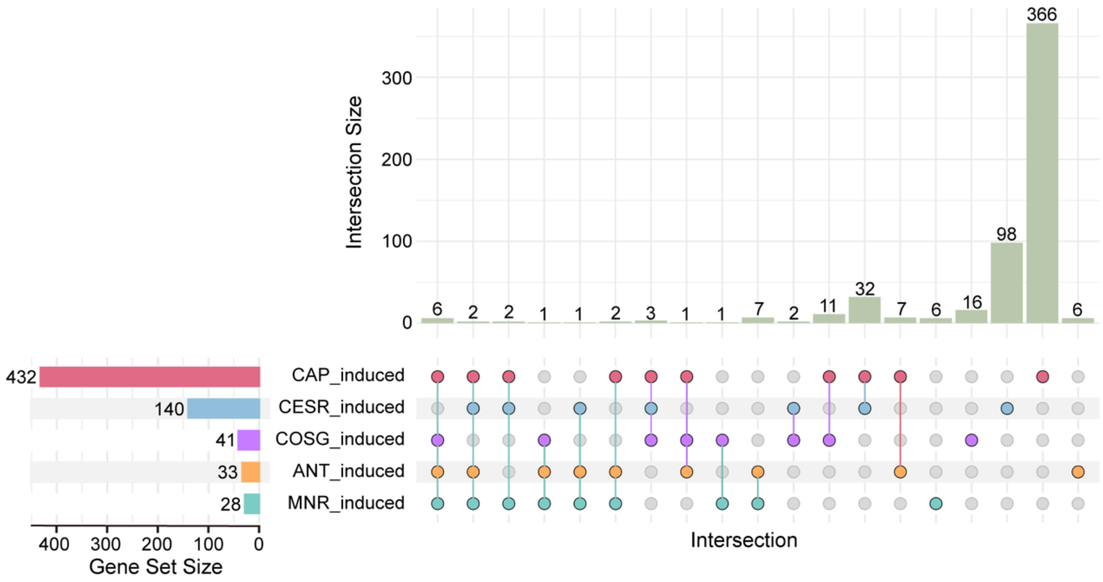

Fig. S3 Overlaps of genes induced in CAP-treated stationary-phase cells grown in glucose medium with genes induced in different stress conditions (as in Fig. 1c). The gene sets include the core environmental stress response (CESR), core oxidative stress genes (COSG), antimycin A treatment (ANT), and the mito-nuclear retrograde (MNR) response. The gene set size (left) indicates the number of genes in each set. Each column of circles represents a specific intersection between the different gene sets, as indicated by the colored circles. Bars (top) indicate the corresponding numbers of genes for each intersection. The plot was generated using *ComplexUpset* package in R (version 4.4.1, R Foundation for Statistical Computing, Vienna, Austria).

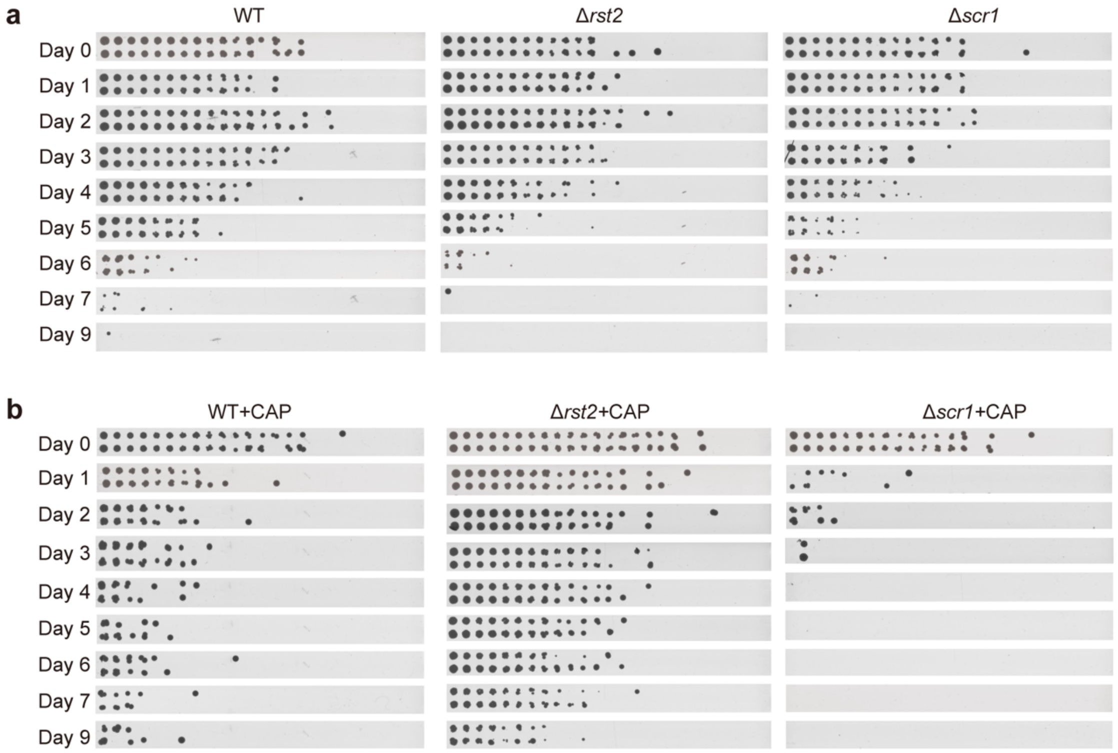

Fig. S4 Chronological lifespan assays. Wild type, Δ*rst2* and Δ*scr1* cells were precultured overnight in YES medium at 32°C, diluted to an initial OD_{600} of 0.02 and grown to stationary phase (Day 0) without (**a**) or with (**b**) CAP treatment. Cell viability was monitored over time by a robotics-based colony-forming unit (CFU) assay. CFUs were measured daily from Day 0 to Day 7 and at Day 9. At each time point, aliquots of ageing cultures were collected, 3-fold serially diluted, and spotted onto YES agar plates. Plates were incubated at 32°C for 3 days and subsequently imaged. Three independent biological replicates were prepared for each condition.

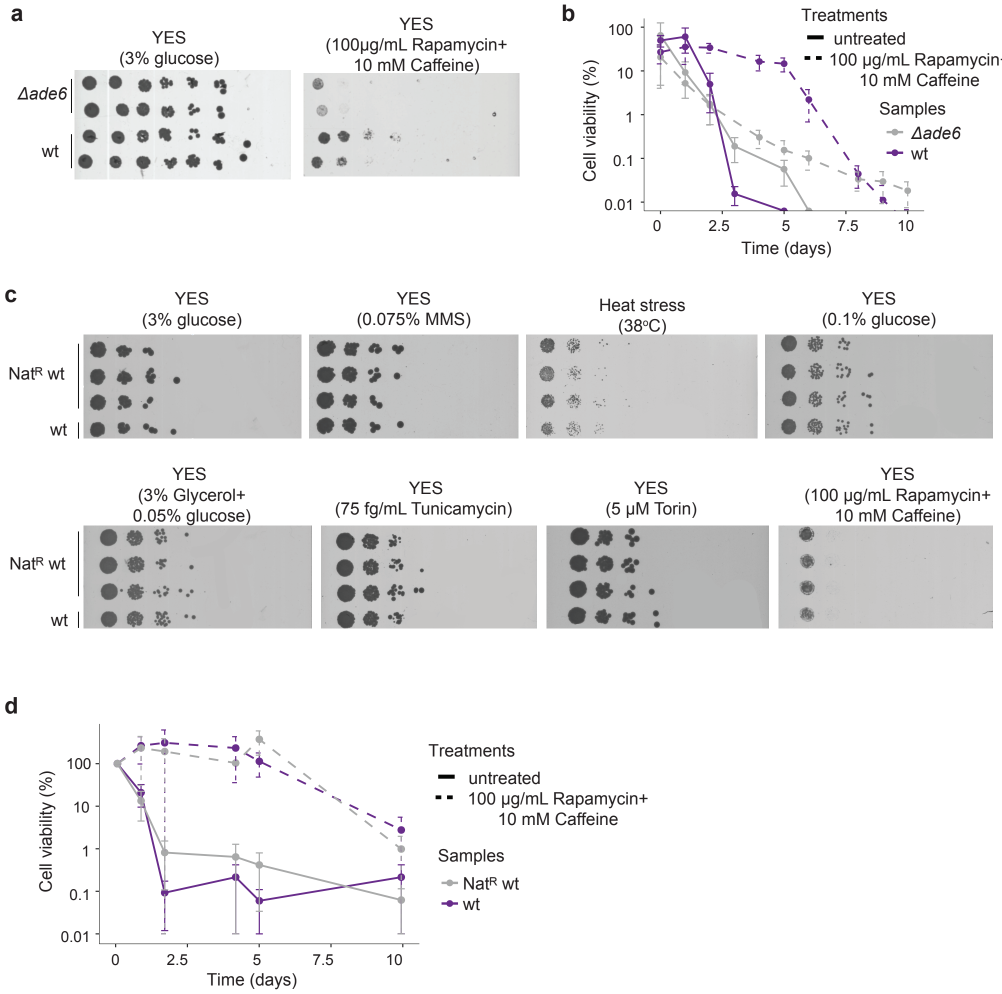

Fig. S5 The Nat^{R} wild-type strain, unlike the *Δade6* strain, shows no phenotypic differences compared to the original wild-type. (**a**) Spot assays comparing Δ*ade6* and wild-type cells on YES (3% glucose) and YES (3% glucose) supplemented with 100 µg/mL rapamycin + 10 mM caffeine. Cultures were adjusted to OD_{600} = 0.2, serially diluted 1:3, and spotted (left to right) onto the indicated plates. Plates were incubated at 32°C for 2-3 days. (**b**) Chronological lifespan assay of wild-type and Δ*ade6* strains with or without 100 µg/mL rapamycin + 10 mM caffeine. Cells were grown in YES to stationary phase (Day 0). Viability was assessed over time using a robotics-based colony-forming unit (CFU) assay, with values normalized to Day 0 (100%). At each time point, ageing cultures were serially diluted 1:3, spotted onto YES plates, and imaged after three days. Data represent three biological replicates; error bars show standard deviation (SD). (**c**) Spot assays comparing Nat^{R} wild-type and the original wild-type under: DNA damage (0.075% MMS), heat stress (38°C), low glucose (0.1%), non-fermentable carbon source (3% glycerol + 0.05% glucose), ER stress (75 fg/mL Tunicamycin), TOR inhibition (5 µM Torin), and Rapamycin + Caffeine (100 µg/mL + 10 mM). Cultures were adjusted to OD_{600} = 0.2, serially diluted 1:10, and spotted and incubated as above. (**d**) Chronological lifespan assay of Nat^{R} wild-type and wild-type cells with or without rapamycin + caffeine. Assay was carried out as described in (b) above.

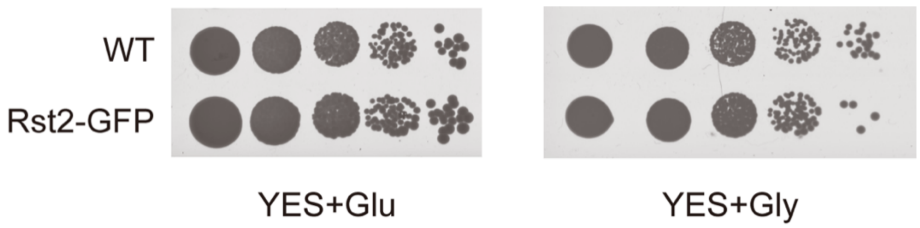

Fig. S6 Growth assessment of the Rst2-GFP strain. The strain expressing C-terminally GFP-tagged Rst2 from its endogenous promoter was precultured overnight, diluted to an initial OD_{600} of 0.2, and grown to OD_{600} of 1. Cells were then collected, and 10-fold serial dilutions were spotted onto YES plates containing either 3% glucose or 3% glycerol supplemented with 0.1% glucose. The plates were incubated at 32℃ for 3 days and photographed.
