# Supplementary figures and images for: Mitochondrial Translation Inhibition Triggers an Rst2-Controlled Transcriptional Reprogramming of Carbon Metabolism in Stationary-Phase Cells of Fission Yeast
Source: Biomolecules. 2025 Sep 24;15(10):1354. doi: 10.3390/biom15101354 (PMC12564598; doi:10.3390/biom15101354)

Fig. 5e

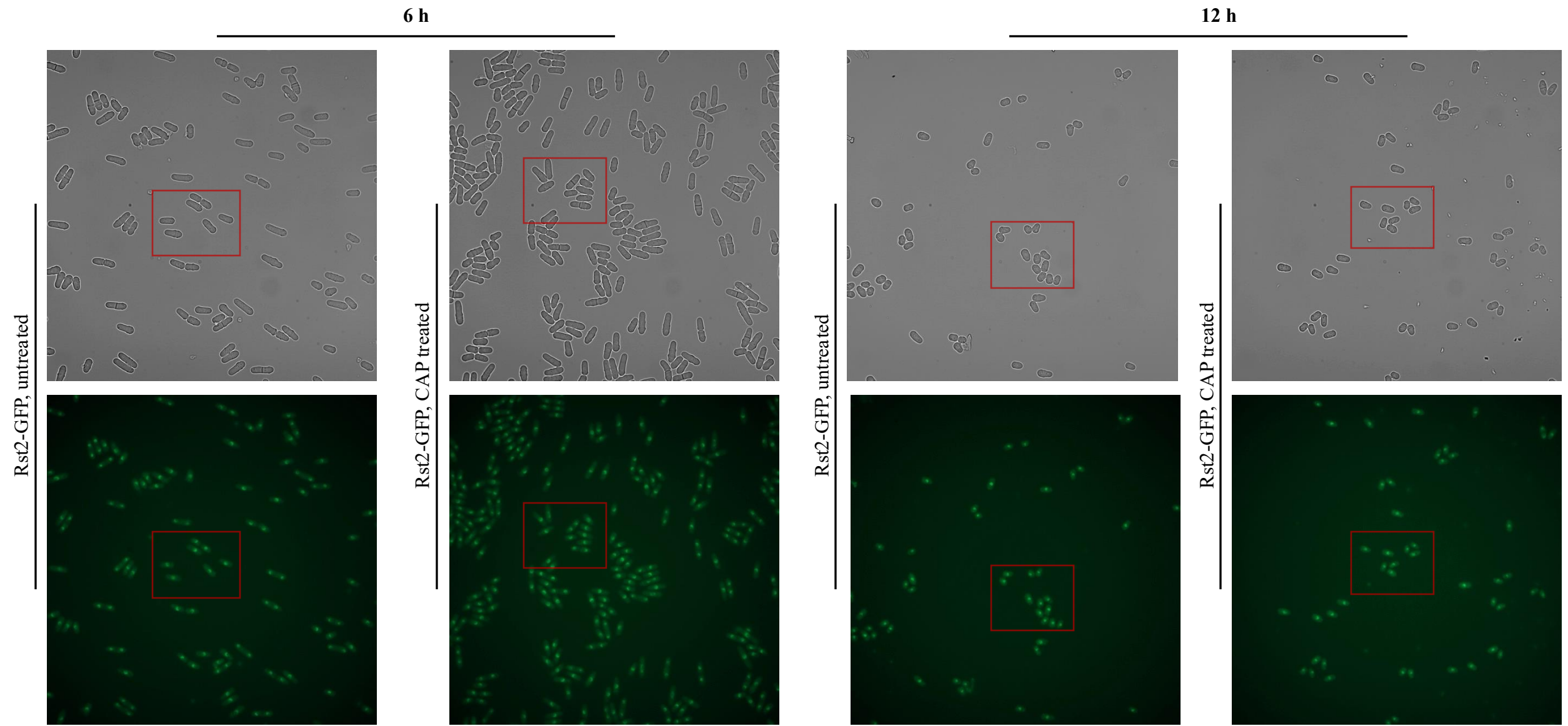

Fig. 5e

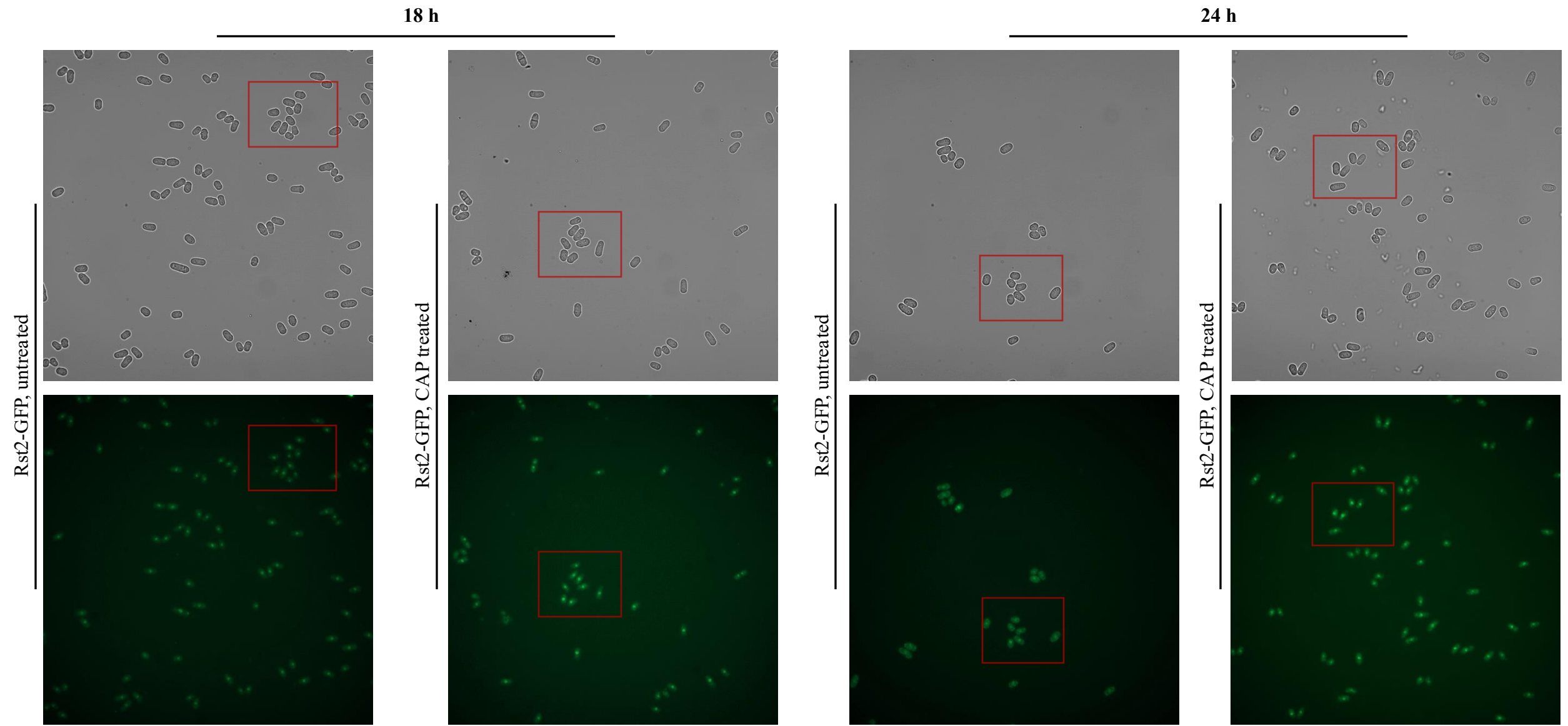

Fig. 5e

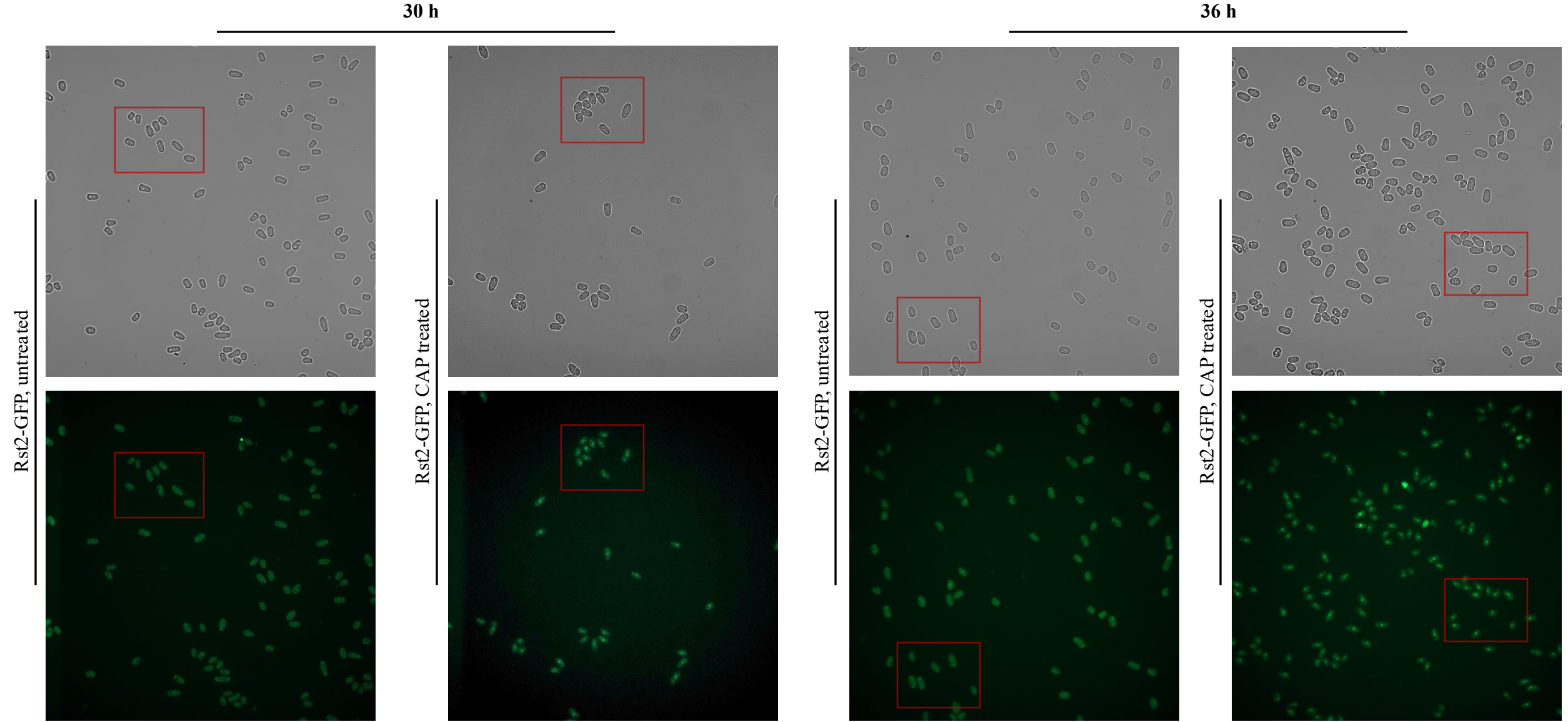

Supplement: Supplementary file 1 [file biomolecules-15-01354-s001.zip › Supplementary File S1 Original Microscopy Images.pdf]
